# Diet Breadth Mediates the Prey Specificity of Venom Potency in Snakes

**DOI:** 10.3390/toxins12020074

**Published:** 2020-01-23

**Authors:** Keith Lyons, Michel M. Dugon, Kevin Healy

**Affiliations:** Ryan Institute, School of Natural Sciences, National University of Ireland Galway, H91 R8EC Galway, Ireland; michel.dugon@nuigalway.ie

**Keywords:** venom, snakes, LD_50_, potency, prey-specific, phylogenetic comparative analyses, predator, diet, phylogenetic comparative biology, diet breadth

## Abstract

Venoms are best known for their ability to incapacitate prey. In predatory groups, venom potency is predicted to reflect ecological and evolutionary drivers relating to diet. While venoms have been found to have prey-specific potencies, the role of diet breadth on venom potencies has yet to be tested at large macroecological scales. Here, using a comparative analysis of 100 snake species, we show that the evolution of prey-specific venom potencies is contingent on the breadth of a species’ diet. We find that while snake venom is more potent when tested on species closely related to natural prey items, we only find this prey-specific pattern in species with taxonomically narrow diets. While we find that the taxonomic diversity of a snakes’ diet mediates the prey specificity of its venom, the species richness of its diet was not found to affect these prey-specific potency patterns. This indicates that the physiological diversity of a species’ diet is an important driver of the evolution of generalist venom potencies. These findings suggest that the venoms of species with taxonomically diverse diets may be better suited to incapacitating novel prey species and hence play an important role for species within changing environments.

## 1. Introduction

Venoms have evolved through natural selection into complex mixtures, comprised of diverse assortments of toxic proteins, peptides, minerals and molecular components [1,2,3,4]. When delivered into a target organism, these components interact to create a functional response, typically to subjugate prey or deter threats [5,6,7,8,9]. While venom has evolved in response to several factors [10,11,12], for species that use venom for predation, various functional aspects of their venoms, such as their potency, are expected to reflect the evolutionary pressures related to capturing prey [5]. In particular, as the functional ability of such venoms are selected through their efficacy against the organisms they are most frequently targeted towards, they are expected to show patterns of prey-specific potencies [13,14]. Examples of venoms which show such prey-specific potency are found across the animal kingdom, including spiders [13,15,16,17], cone snails [11,18,19], centipedes [20], heteropterans [21] and snakes [22]. Furthermore, in snakes, prey-specific potencies seem to be the rule rather than the exception, with increasing potencies found to be associated with venoms tested on animals more closely related to the snakes’ diet [14,23,24]. However, despite the apparent prevalence of prey-specific venoms, there are many cases of venoms that do not show prey-specific patterns [25,26,27], and little is known regarding how the complexity of a predator’s diet may mediate known potential prey-specific patterns.

In the simplest case, the venom from a predator which preys on a single species will be prey-specifically selected against that given species [13]. However, predators typically have diets comprising multiple species, often from a range of taxonomic groups. The selection pressures on species with these diverse diets are hence likely to be more complex, with such venoms under evolutionary pressures to be functional against a range of different species and physiologies. Hence, the venom of predators with specialist diets are predicted to show higher degrees of prey-specific potency in comparison to those with generalist diets [13]. Evidence for such mediating effects of diet diversity on prey specificity has been shown in cone snails (*Conus* sp.), where species with more diverse diets were found to have more complex venoms, potentially allowing them to target a broader range of prey species [18,28]. Similarly, in a study of the venom efficacy between the preferred and alternative prey of twenty-two arachnid species, species with specialised diets were found to have more pronounced prey-specific potencies compared to generalist feeders [13]. However, despite the potential importance of diet breadth, large comparative analyses on the role of diet breadth in venom evolution are lacking, in particular, in otherwise well-studied groups such as snakes.

Snake venoms have evolved in a wide diversity of ecologies, ranging from the specialist fish–egg diet of the Marbled sea snake (*Aipysurus eydouxii*) [29] to the diverse opportunistic diet of the King brown snake (*Pseudechis australis*) [30]. This wide range of ecologies representing different potential selection pressures make snakes an ideal system on which to test the role of trophic ecology on the evolution of predator traits. Previous studies have used snakes as a group to test the role of trophic ecology in driving venom composition [31,32,33,34], potency [24,35,36,37], and the evolutionary atrophy of venom systems [38]. However, the use of non-standardised test models which typically do not represent natural prey species have previously confounded comparative analysis of venom potency [24,39,40,41,42,43]. One approach to account for this confounding issue is to include a measure of the evolutionary distance between the natural prey species and the species on which a venom potency has been measured [14]. This approach allows venom potency data measured on a wide range of animals to be used to test hypothesises relating to the functional evolution of venom within standard phylogenetic comparative frameworks [14]. Here, we extend on previous comparative analysis of snake venom [14] by testing the role of diet breadth in mediating the prey specificity of venom potency.

**Figure 1:**
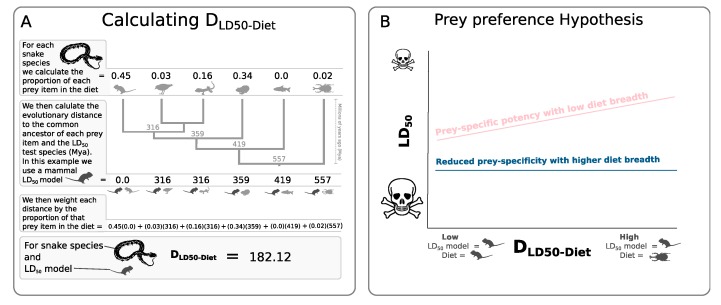
(**A**) Example of the calculation of the mean phylogenetic distance between the LD_50_ potency-model species and the prey species of a snake. (**B**) Predictions of the prey-specific patterns of snakes with low (pink) and high (blue) diet breadth. Prey-specific venoms are expected to show decreasing potencies (higher LD_50_) measured on model species more phylogenetically distance from their prey (high D_LD50-Diet_). Under the prey preference hypothesis, a prey-specific pattern is expected for species with low diet breadth (pink line), while such prey species patterns are expected to be reduced in species with wider diet breaths (blue line).

Like an ecological community, a predator’s diet can be diverse in numerous ways. For example, while the diet of the Western diamondback rattlesnake (*Crotalus atrox*) is species rich, much of its diet is comprised of rodent species [44]. In contrast, while the diet of a King brown snake (*Pseudechis australis*) may have a similar species richness to that of the Western diamondback rattlesnake, the diversity of its prey ranges from taxonomically disparate groups including amphibians, mammals, birds and invertebrates [30]. From an evolutionary perspective, the venom of a snake with a phylogenetically restricted diet is unlikely to experience the same selection pressures compared to a venom that is required to incapacitate prey from various different taxonomic groups. Here we use various measures of diet diversity, including species richness, taxonomic richness and phylogenetic diversity [45], to test the role of diet breadth in mediating the prey-specific nature of venom potency in snakes. We predict that snake species with taxonomically or phylogenetically narrow diets will have venom potencies displaying the strongest prey-specific effects (Figure 1). Prey-specific venoms are expected to show decreasing potencies, with increasing evolutionary distances between potency-model species and natural prey species (D_LD50-Diet_) [14]. Hence, we hypothesise that diet breadth will interact negatively with D_LD50-Diet_, so that species with narrow diets show stronger prey-specific potencies when compared to species with more generalist diets.

## 2. Results

Our dataset consisted of 100 snake species, with 529 measures of LD_50_. Values for LD_50_ ranged from 1121 mg kg^−1^ for the Western diamondback rattlesnake (*C. atrox*) when tested on the Virginia opossum (*D. virginiana*) to 0.00031 mg kg^−1^ in the many-banded krait (*B. multicinctus*) when measured on the White-rumped munia (*L. striata*). Mammals comprised the majority of potency-model species in the dataset (79.6% of all measures), with mice (*Mus musculus*) comprising 77.6% of all measures. The remaining LD_50_ values collated from the literature were estimated from fish (6.8%), reptiles (6.4%), birds (5.9%), amphibians (0.9%) and arthropods (0.4%).

**Figure 2:**
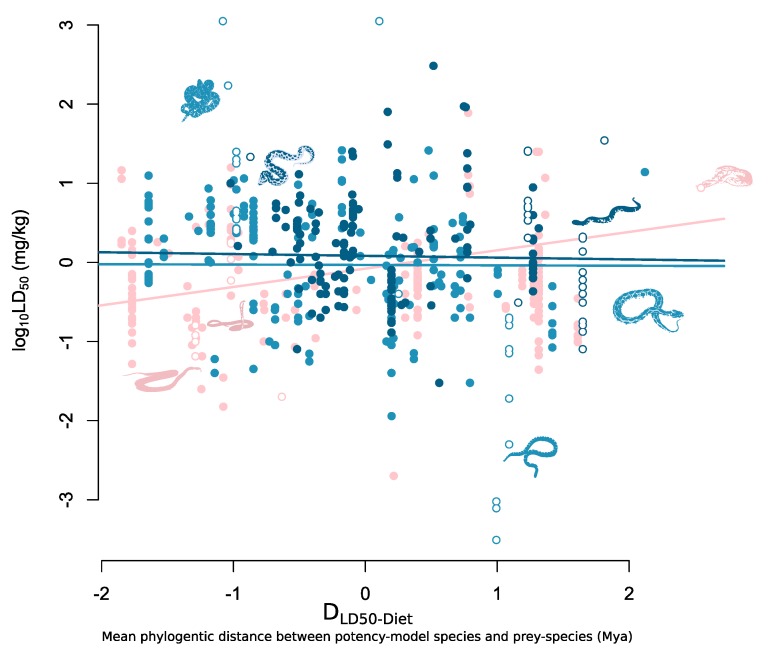
Mean phylogenetic distance between potency-model species and natural prey (D_LD50-Diet_) against log_10_ LD_50_. Species with only a single taxonomic group represented in their diets are represented by the pink points and fitted line (intercept = 0.33, slope = 0.22); species with an intermediate diversity of taxonomic groups represented in their diets are represented by the blue points and fitted line (intercept = 0.39, slope = 0.00); and species with high taxonomical diverse diets are represented by the dark blue points and fitted line (intercept = 0.45, slope = −0.03). Hollow points represent silhouette species which are, from left to right: *Oxyuranus scutellatus*; *Crotalus* *adamanteus*; *Ophiophagus hannah*; *Vipera latastei*; *Agkistrodon piscivorus*; *Bungarus multicinctus; Daboia russelii*; *Causus rhombeatus*.

In our analyses, we found support that the taxonomic richness of a snakes’ diet mediates the prey-specific potency of its venom, with a negative interaction term between D_LD50-Diet_ and diet breadth, indicating that snake species with taxonomically narrow diets have venom potencies that are more prey-specific (Table 1, Figure 2).

In all models, the LD_50_ of a snakes’ venom was associated with the route that it was administrated into the test model, the presence of eggs in the diet, and by the mean phylogenetic distance between the potency-model species and natural prey species (D_LD50-Diet_; Table 1). Lower LD_50_ values, and hence higher potency, was found when venom was administered via intravenous (IV) or intraperitoneal (IP) routes when compared to subcutaneous (SC) routes (Table 1). As found in previous analyses, species which have eggs present in their diets are associated with lower venom potencies in all models (Table 1). The mean phylogenetic distance between potency-model species and natural prey (D_LD50-Diet_) was also found to have a positive effect in all models, indicating that venom potency decreases when tested in species phylogenetically distant from natural prey species (Table 1).

In the model using taxonomic richness as a measure of diet breadth we found that there was a negative interaction term between taxonomic richness and D_LD50-Diet_ (Table 1, Figure 2). We found that the negative effect size associated with the interaction term for both species with intermediate taxonomic richness (β = −0.22, lower 95% CI = −0.40, upper 95% = −0.07) and high taxonomic richness (β = −0.25, lower 95% CI = −0.48, higher 95% = −0.01) was equal or larger than the affect size associated with D_LD50-Diet_ (β = 0.22, lower 95% CI = 0.12, upper 95% = 0.33). This resulted in species with diets comprising more than one taxonomic group showing no prey-specific potency relationship between LD_50_ and D_LD50-Diet_ (Figure 2). While we find similar negative effects in the interaction terms in the model using species richness and phylogenetic distance as measures of diet breadth, the upper 95% credibility intervals (CI) of the posterior distributions in both models was greater than zero, hence reducing our confidence in rejecting a null relationship in these models (Table 1).

While the interaction term between D_LD50-Diet_ and diet breadth was found to be associated with LD_50_, the fixed term for diet breadth itself was not found to have any effect on LD_50_ in any model, showing that species with specialised or generalist diets do not show differing levels of venom potency in general (Table 1). In all models’ phylogenetic signals between 0.42 and 0.47 were found indicating that while LD_50_ is phylogenetically conserved to some degree it is relatively pliable over the evolutionary scale represented by the species in the analysis (Table 1).

In our supplementary analysis we find qualitatively similar results to our main analysis with a significant negative interaction term between taxonomic richness and D_LD50-Diet_ found in all models (Supplementary Materials Tables S1–S6). While we find a significant negative interaction for either intermediate or high taxonomic richness in all models (Supplementary Materials Tables S1–S6), the Upper 95% CI of the posterior distribution is above zero for the interaction term associated with the high taxonomic richness category in the models that include mean prey size (β = −0.24, Lower CI = −0.53, Upper CI = 0.08, Supplementary Materials Table S3), LD_50_ experiment duration (β = −0.26, Lower CI = −0.55, Upper CI = 0.01, Supplementary Materials Table S4) and Sensitivity Analysis 2 model (β = −0.25, Lower CI = −0.50, Upper CI = 0.01, Supplementary Materials Table S6) and for the interaction term associated with intermediate taxonomic richness in the model that includes whether LD_50_ models were captive or wild (β = −0.13, Lower CI = −0.30, Upper CI = 0.02, Supplementary Materials Table S5). We find no association between LD_50_ and the proportion of endothermic prey in the diet (Supplementary Materials Table S1), the presence of constriction behaviour (Supplementary Materials Table S2); mean prey size (Supplementary Materials Table S3) and the LD_50_ experiment duration (Supplementary Materials Table S4). The use of wild species to measure LD_50_ was found to have a positive association with LD_50_, indicating that the use of captive bred animals is associated with high potencies compared to wild test subjects (Supplementary Materials Table S5).

## 3. Discussion

Our findings show that while venom potency is prey-specific, this prey specificity is mediated by the breadth of the snake’s diet, with snakes with taxonomically diverse diets having less prey-specific venom potencies. In snakes with taxonomically narrow diets, venom potencies were found to decrease with larger evolutionary distances between potency-model species and natural prey species (D_LD50-Diet_). This prey-specific pattern follows the expected pattern that venoms are selected to have the highest functional efficacy against common targets, such as seen in several predator–prey cases studies, prey switching experiments [13,22,31,39,46], and comparative analysis [14]. However, we find that this prey-specific relationship is lost in species with more than one taxonomic class of prey in their diets, as indicated by a negative interaction between diet breadth and D_LD50-Diet_. These results support the prey preference hypothesis [13], where the venoms of species with taxonomically diverse prey are selected to have less prey-specific potencies in comparison to specialist feeders. This difference in the levels of prey specificity of venom is expected as, while the venoms of more specialist feeders, such as King cobras (*Ophiophagus hannah*), are likely to be selected to incapacitate a relatively narrow range of physiologies, the venoms of generalist feeders are likely to be selected to have the ability to incapacitate a larger diversity of physiologies, such as the case in King brown snakes (*Pseudechis australis*), which prey on species ranging from invertebrates to mammals [30].

This mediating role of diet breadth in the prey specificity of venoms is supported by prey switching experiments and in the patterns of venom complexity found in several animal groups [13,18,28,47]. As the venoms of generalist feeders are selected to be active against a wide variety of organisms, these venoms are expected to be more complex in order to have the capacity to target a large range of physiologies. Such a pattern is observed in the lack of complexity in sea snake venoms, which primarily feed on fish, when compared to snakes with more generalist diets [9,29]. Furthermore, in a more recent comparative analysis, the toxicological diversity of snake venoms was found to be higher in species with a more generalist diet [48]. Similarly, in cone snails, venom complexity is found to increase with diet breadth, suggesting that the relationship between diet breadth and venom complexity may be a general pattern found across venomous taxonomic groups [18,28]. However, while venom complexity and composition may be indicative of the selective role of diet breadth, it is the resulting functional responses of these venoms on potential prey, such as the ability to incapacitate, that is expected to be under the strongest selection pressures. One analysis which used paralysis latency as a measure of venoms functional ability in spiders found that in a prey switching experiment, the venoms from species with specialised diets had reduced potencies when tested on novel prey, while spiders with more generalist diets did not [13]. Our results extend the support for this diet breadth mediated pattern of venom specificity, both in terms of extending it to snakes and in using a phylogenetic comparative framework.

While we found strong support for the mediating role of taxonomic richness in prey-specific potencies, we found weaker support when using species richness or prey diversity as a measure of diet breadth. This suggests that disparity of prey physiology is a key aspect in the relationship between diet breadth and the evolution of prey specificity in snake venoms. As taxonomic divisions are classically designated based on morphological and physiological differences, taxonomic richness is more likely to capture the range of physiologies in a snake’s diet compared to species richness or phylogenetic diversity. For example, the phylogenetic distance between groups of fish can often be higher than between groups of physiologically distinct amniotes [49]. Hence, the phylogenetic diversity of the diet of the Olive sea snake (*Aipysurus laevis*), which primarily feeds on fish, is higher in our analysis than the diet of the Common death adder (*Acanthophis antarcticus*), which feeds on mammals, reptiles, birds and amphibians [9,50]. This potential importance of prey physiological disparity has been highlighted as an explanation for the comparatively simple venoms of sea snakes when compared to their more generalist feeding terrestrial counterparts [47] and for the increased toxicological diversity of venoms associated with species with more taxonomically diverse diets [48]. Furthermore, in *Micrurus* coral snakes [39] and in Black widow spiders [51], venoms have also been found to show compositional structures defined along the taxonomic grouping of their prey. For example, members of the genus *Latrodectus* produce at least seven types of latrotoxins in order to target the insect, vertebrate and crustacean taxonomic groups found it their diets [51]. However, how the composition of venoms map onto the physiological disparity of their diets is likely to be complex. For example, venoms have been found to have substantially different potencies even when tested on prey with similar physiologies [52]. Furthermore, while broader diet breadth has been found to be associated with more complex venoms in both cone snails [28] and snakes [48], associations between specific taxonomic prey groups and particular molecular or toxicological aspects of venoms have been either absent or weak. Hence, while diverse diets are associated with more complex venoms the mechanistic pathways these venoms achieve such diverse functionality is still not clear.

The association between diet and venom is likely to be complex. For example, a recent study of rattlesnakes has found that other factors, and not diet, were the main drivers of venom variation [27]. This highlights the need for further studies testing the role of other potential drivers of venom potency, such as diet seasonality [53] and environmental factors [27]. Furthermore, the use of lethality as a measure of potency may overlook important selection pressures on venom. Venoms are expected to be under selection to incapacitate prey and while inducing mortality will incapacitate prey, the speed at which a venom can act or the ability to stun prey are also likely to be important functional aspects of venom. While the taxonomic coverage of LD_50_ measures allows for its use in large comparative analysis, its insights into the various functional aspects of venoms are likely to be limited. Other measures of venom potency, such as paralysis latency or median effective dose (ED_50_), will aid in identifying the fundamental drivers of venom functional evolution. For example, measures of the speed of incapacitation have been used to show prey-specific patterns in *Echis* vipers [24]. However, to determine whether this is a general pattern for snake venom will require a larger coverage of snake species and taxonomic groups. Identifying and testing the general patterns between ecological factors and venom functionality and composition at large taxonomic scales can offer a key pathway to understanding the fundamental mechanisms driving the evolution of venom diversity. Ultimately, understanding such mechanisms may contribute to clinical developments [12,54] and aid in bioprospecting [5,55]. From an ecological perspective, understanding the association between diet and venom may aid in our ability to predict the ecological role of venomous predators in rapidly changing ecosystems [56]. For example, the potential invasiveness of certain venomous species may be mediated by the prey specificity of their venoms, with species with generalist venoms, such as the highly invasive Brown widow (*Latrodectus geometricus*) and the Noble false widow (*Steatoda nobilis*), more likely to have venoms which can functionally take advantage of novel prey items [57,58]. Developing clear ecological and evolutionary predictions regarding the composition and functionality of venoms across the animal kingdom can not only offer pathways to understanding nature’s complex cocktails, but may also aid in to our ability to manage future medical [5,54,55] and ecological issues relating to venom [5].

## 4. Materials and Methods

To test the evolutionary drivers of venom potency, we used potency and dietary data previously collated in Healy et al., [14]. This data used median lethal dose (LD_50_) as a measure of venom lethality and included only intravenous (IV), subcutaneous (SC), intraperitoneal (IP) or intramuscular routes (IM) of administering venom.

To test for prey-specific patterns in venom potency we used the mean phylogenetic distance, measured as divergence time (Millions of years ago (Mya)), between the LD_50_ potency-model species and the natural prey species of the snake. This mean phylogenetic distance (D_LD50-Diet_) was calculated as D_LD50-Diet (jk)_ = $\sum^{\;}p_{\mathrm{ij}}d_{\mathrm{ik}}$, where D_LD50-Diet (*jk*)_ is the weighted phylogenetic distance between the prey of a focal snake species j and the potency-model species *k*, $p_{i}$ is the proportion of snake species *j*s diet that is comprised by prey item i and $d_{ik}$ is the phylogenetic distance, measured as the divergent distance to the common ancestor of both prey item i and the potency-model species k. D_LD50-Diet (*jk*)_ hence gives the mean phylogenetic distance between each prey item *i* and the poteceny-model species *k* weighted according to the proportion of each prey species in the snake’s diet. Phylogenetic distances were calculated using published phylogenies for mammals [59], squamates [60], amphibians [61] and fish [49] for prey items identified to genus or species levels. TimeTree [62] was used for prey items only identified to family level or above. As the presence of eggs in a species’ diet has previously been found to be associated with reduced potencies [14,29], we also included a separate term indicating the presence/absence term in our analysis.

Dietary data was collated from the literature using studies with quantitative estimates of prey proportions, mainly from studies of stomach contents [14]. Using this data, we calculated the species richness, taxonomic richness and phylogenetic richness of the diet for each snakes’ species. Species richness was calculated as the number of prey items that could be separated into distinct taxonomic categories. For example, a diet comprised of three prey items from three separate taxonomic groups was assigned a species richness of three, even if each prey item was not identified to species level. Taxon richness was defined by the number of distinct taxonomic groups found in the diet. Following previous analyses of snake diets [63], we used the taxonomic class breakdown of Mammalia, Aves, Reptiles, Amphibia, Fish and Invertebrates. Snake species with diets comprising only one taxonomic group were defined as having low taxonomic richness (low), those with diets comprising between two and three taxonomic groups as having intermediately broad diets (intermediate) and those with over three as having taxonomically broad diets (high). Phylogenetic diversity is a metric used in community ecology to incorporate the evolutionary distinctness of species in a given community [64]. We calculate phylogenetic diversity by building a phylogeny of the identifiable items in each diet and using the picante package [65] to calculate Faith’s PD, the sum of the total phylogenetic branch length, for each diet as it represents the alpha diversity of the species within the diet [45]. We used the mean prey size data calculated in Healy et al., [14]. We used data on the presence of constriction behaviour from Healy et al., [14], which was primarly collated from Shine and Schwaner [66]. As venom yield was not found to correlate with LD_50_ in previous analyses [14], we did not include it here. All data is available in the Supplementary Materials Dataset S1.

## 5. Analysis

Variables were log_10_ transformed, mean centred and expressed in units of standard deviation. This allowed effect sizes of variables expressed across different scales to be compared within the same model. To test our predictions on the role of diet breadth in mediated prey-specific potency, we fitted Bayesian multivariate phylogenetic mixed models using the MCMCglmm package [67] in R version 3.5.2 [68]. We chose the MCMCglmm package as, through the use of random effects, it allows for the inclusion of both multiple LD_50_ measures for each species and phylogenetic effects. We fit the main model with LD_50_ as the response variable and LD_50_ inoculation method (SC, IM, IV, IP); the presence of eggs in the diet (absent, present); diet breadth (DB); phylogenetic distance of diet species to LD_50_ model (D_LD50–Diet_), and an interaction term between D_LD50-Diet_ and DB as the explanatory variables. This gave use the general model;

LD_50_ = *f*(LD_50_ method + DB + D_LD50-Diet_ + DB: D_LD50-Diet_) (529 observations, 100 species) (1)

In each model, non-independence of data due to common descent was controlled by including the phylogeny [60] using the animal term in the MCMCglmm model. Variation due to multiple measures on individual species was also accounted for using a separate random term. The relative variance attributable to the phylogenetic random effect component (h^2^) was calculated as the ratio of variance explained by phylogeny to the sum of phylogenetic variance, species variance and residual variance [69]. All models were fitted with parameter expanded priors. The burn-in, thinning and number of iterations were determined for each model separately to ensure effective sample sizes exceeded 1000 for all parameter estimates. We tested for convergence using the Gelman-Rubin statistic over three separate chains [70]. The significance of each variable’s effect on LD_50_ was determined based on whether the 95% credibility interval of the variables posterior distribution was either below or above zero.

We ran three separate models using each of the three measures of diet breadth; species richness, taxonomic richness and phylogenetic diversity. If diet breadth mediates prey-specific potency, we predict a significant negative interaction term between DB and D_LD50-Diet_. This would indicate that species with more specialised diets have a stronger increase in LD_50_, and hence decrease in potency, with increasing phylogenetic distances between the LD_50_ test model and the diet (D_LD50-Diet_).

To account for various potential confounding factors, we ran a series of supplementary analyses. As venom potency may be related to the metabolic state of prey species, we ran the main analysis as outlined above with the proportion of endothermic prey in the diet of each snake species (calculated as the proportion of Mammalia and Aves prey items) included as an explanatory term. As species with constriction may not rely on venom to incapacitate prey, we ran supplementary models with an additional explanatory term of whether constriction behaviour has been observed for a species. To account for the potential role of prey size in driving venom LD_50_, we used previously collated mean prey size data and ran an additional set of models with mean prey size as an additional explanatory factor. As LD_50_ experiments with longer durations may result in higher measures of potency, we included the reported experiment duration length as an explanatory factor in additional supplementary models. To account for potential variation associated with experiments using either captive bred or wild caught test animals, we included an explanatory variable with two levels (captive, wild) in a set of additional analysis. Finally, we ran two sets of sensitivity analysis for the low, intermediate and high categories used for diet taxonomic richness, one analysis with taxonomic richness split as low (1 taxonomic group), intermediate (2–3 taxonomic groups) and high (more than 3 taxonomic groups) and a separate analysis with taxonomic richness split as low (1 taxonomic group), intermediate (2–4 taxonomic groups) and high (more than 4 taxonomic groups).

## Figures and Tables

**Table 1:** Main models testing the role of three different measures of diet breadth on LD_50_. Modes (β) and 95% credibility intervals (Lower CI and Upper CI) of the posterior distributions from the three main models predicting LD_50_ using taxonomic richness, phylogenetic diversity and species richness as measures of diet breadth. Fixed factors include the continuous factors of mean phylogenetic distance between diet species and the LD_50_ model (D_LD50-Diet_) and diet breadth (DB) for models that use phylogenetic diversity and species richness. Categorical fixed factors include LD_50_ method (subcutaneous (SC), intravenous (IV), intraperitoneal (IP) and intramuscular (IM)); the presence of eggs in the diet (Eggs in Diet; present, absent) and the taxonomic richness measure of diet breadth (Low, Intermediate and High). For categorical factors, the baseline was SC for LD_50_ method; the absence of eggs for the Eggs in Diet variable and low for taxonomic richness. DB_Low_:D_LD50-Diet_ represents the interaction term between DB and D_LD50-Diet_. The random terms associated with phylogenetic relatedness (Phylogeny (h^2^)), intraspecific variation (Species) and residual variation (Residual) are also presented. Significant values, which are highlighted in bold, are deemed to be those with 95% of the posterior estimate above or below zero. For more detail on the parameters used, see the Materials and Methods section. All models have 529 LD_50_ measures for 100 species.

	Taxonomic Richness Model			Phylogenetic Diversity Model			Species Richness Model		
	β	Lower CI	Upper CI	β	Lower CI	Upper CI	β	Lower CI	Upper CI
**Fixed Terms**									
Intercept	0.33	−0.15	0.79	0.40	0.03	0.88	0.40	−0.02	0.92
LD_50_ method_SC_									
*IV*	**−0.53**	**−0.67**	**0.38**	**−0.53**	**−0.67**	**−0.39**	**−0.53**	**−0.67**	**−0.38**
*IP*	**−0.26**	**−0.42**	**−0.12**	**−0.26**	**−0.42**	**−0.12**	**−0.26**	**−0.41**	**−0.12**
*IM*	−0.12	−0.29	0.06	−0.12	−0.30	0.06	−0.12	−0.28	0.07
Eggs in Diet									
*Present*	**0.88**	**0.33**	**1.36**	**0.86**	**0.34**	**1.45**	**0.89**	**0.32**	**1.42**
D_LD50-Diet_	**0.22**	**0.12**	**0.33**	**0.10**	**0.02**	**0.18**	**0.11**	**0.02**	**0.18**
Diet Breadth_Low_				0.03	−0.07	0.12	0.01	−0.08	0.-10
*Intermediate*	0.06	−0.19	0.33	-	-	-	-	-	-
*High*	0.12	−0.17	0.44	-	-	-	-	-	
DB_Low_:D_LD50-Diet_				−0.06	−0.13	0.01	−0.05	−0.13	0.01
*Intermediate*	**−0.22**	**−0.40**	**−0.07**	-	-	-	-	-	-
*High*	**−0.25**	**−0.48**	**−0.01**	-	-	-	-	-	-
**Random Terms**									
Phylogeny (h^2^)	0.42	0.12	0.74	0.46	0.21	0.71	0.47	0.22	0.71
Species	0.15	0.01	0.33	0.13	0.01	0.31	0.13	0.01	0.30
Residuals	0.41	0.27	0.53	0.39	0.25	0.52	0.41	0.25	0.51

## Supplementary Materials

The following are available online at https://www.mdpi.com/2072-6651/12/2/74/s1, Dataset S1: Dataset S1, Table S1: Models with proportion of endothermic prey items included as a fixed factor., Table S2: Models with presence of constriction included as a fixed factor., Table S3: Models with mean prey size included as a fixed factor., Table S4: Models with LD50 duration included as a fixed factor., Table S5: Models with the wild/captive status of the LD50 model included as a fixed factor., Table S6: Sensitivity analysis of Taxonomic Richness Models.
