# Supplementary material for: Diet Breadth Mediates the Prey Specificity of Venom Potency in Snakes
Source: Toxins (Basel). 2020 Jan 23;12(2):74. doi: 10.3390/toxins12020074 (PMC7076792; doi:10.3390/toxins12020074)
Supplement: Supplementary file 1 [file toxins-12-00074-s001.zip › toxins-686816-supplementary-resubmit.pdf]

Supplementary Materials: Diet Breadth Mediates the Prey Specificity of Venom Potency in Snakes

Keith Lyons, Michel M. Dugon and Kevin Healy

Table S1. Models with proportion of endothermic prey items included as a fixed factor. Modes (β) and 95% credibility intervals (Lower CI and Upper CI) of the posterior distributions from the three main models predicting LD_{50} using Taxonomic richness, Phylogenetic Diversity and Species Richness as measures of diet breadth. Fixed factors include the continuous factors of mean phylogenetic distance between diet species and the LD_{50} model ($D_{LD50\text{-}Diet}$); the proportion of endothermic prey in the diet (Prey met) and Diet Breadth (DB) in models that use Phylogenetic Diversity and Species Richness. Categorical fixed factors include LD_{50} method (subcutaneous (SC), intravenous (IV), intraperitoneal (IP) and intramuscular (IM)); the presence of eggs in the diet (Eggs in Diet; present, absent) and the Taxonomic Richness measure of Diet Breadth. For categorical factors the baseline was SC for LD_{50} method; the absence of eggs for the Eggs in Diet and low for Taxonomic Richness. DB_{Low}:$D_{LD50\text{-}Diet}$ represents the interaction term between DB and $D_{LD50\text{-}Diet}$. The random terms associated with phylogenetic relatedness (Phylogeny (h^2)), intraspecific variation (Species) and residual variation (Residual) are also presented. Significant values, which are highlighted in bold, are deemed to be those with 95% of the posterior estimate above or below zero. All models have 529 LD_{50} measures for 100 species.

	**Taxonomic Richness Model**			**Phylogenetic Diversity Model**			**Species Richness Model**		
	**β**	**Lower CI**	**Upper CI**	**β**	**Lower CI**	**Upper CI**	**β**	**Lower CI**	**Upper CI**
**Fixed Terms**									
Intercept	0.34	−0.11	0.80	0.41	−0.01	0.88	0.41	−0.05	0.88
LD_{50} $method_{SC}$									
IV	**−0.52**	**−0.66**	**−0.38**	**−0.52**	**−0.66**	**0.37**	**−0.52**	**−0.67**	**−0.38**
IP	**−0.26**	**−0.41**	**−0.11**	**−0.25**	**−0.41**	**−0.10**	**−0.26**	**−0.40**	**−0.12**
IM	−0.12	−0.31	0.04	−0.11	−0.28	0.07	−0.11	−0.29	0.05
Eggs in $Diet_{abscent}$									
Present	**0.90**	**0.38**	**1.40**	**0.88**	**0.36**	**1.40**	**0.89**	**0.42**	**1.49**
$D_{LD50\text{-}Diet}$	**0.23**	**0.13**	**0.34**	**0.12**	**0.03**	**0.21**	**0.13**	**0.04**	**0.21**
Diet $Breadth_{Low}$				0.03	−0.06	0.12	0.02	−0.07	0.11
Intermediate				–	–	–	–	–	–
High				–	–	–	–	–	–
DB_{Low}:$D_{LD50\text{–}Diet}$				−0.06	−0.13	0.01	−0.05	−0.12	0.01
Intermediate	**−0.22**	**−0.38**	**−0.06**				–	–	–
High	**−0.23**	**−0.47**	**−0.01**				–	–	–
Prey met	0.09	−0.09	0.24	0.11	−0.04	0.27	0.12	−0.04	0.30
**Random Terms**									
Phylogeny (h^2)	0.38	0.15	0.68	0.42	0.17	0.69	0.43	0.18	0.70
Species	0.16	0.01	0.34	0.15	0.01	0.34	0.15	0.01	0.33
Residuals	0.43	0.27	0.54	0.41	0.26	0.54	0.40	0.26	0.53

Table S2. Models with presence of constriction included as a fixed factor. Modes (β) and 95% credibility intervals (Lower CI and Upper CI) of the posterior distributions from the three main models predicting LD_{50} using Taxonomic richness, Phylogenetic Diversity and Species Richness as measures of diet breadth. Fixed factors include the continuous factors of mean phylogenetic distance between diet species and the LD_{50} model ($D_{LD50\text{-}Diet}$) and Diet Breadth (DB) in the case of Phylogenetic Diversity and Species Richness. Categorical fixed factors include LD_{50} method (subcutaneous (SC), intravenous (IV), intraperitoneal (IP) and intramuscular (IM)); the presence of eggs in the diet (Eggs in Diet; present, absent); the presence of constriction behavior (Constriction) and the Taxonomic Richness measure of Diet Breadth. For categorical factors the baseline was SC for LD_{50} method; the absence of eggs for the Eggs in Diet; the presence of constriction behavior (Constriction) and low for Taxonomic Richness. DB_{Low}:$D_{LD50\text{-}Diet}$ represents the interaction term between DB and $D_{LD50\text{-}Diet}$. The random terms associated with phylogenetic

relatedness (Phylogeny (h^2)), intraspecific variation (Species) and residual variation (Residual) are also presented. Significant values, which are highlighted in bold, are deemed to be those with 95% of the posterior estimate above or below zero. All models have 529 LD_{50} measures for 100 species.

	**Taxonomic Richness Model**			**Phylogenetic Diversity Model**			**Species Richness Model**		
	**β**	**Upper CI**	**Upper CI**	**β**	**Lower CI**	**Upper CI**	**β**	**Lower CI**	**Upper CI**
**Fixed Terms**									
Intercept	0.35	−0.11	0.84	0.31	−0.21	0.79	0.31	−0.18	0.81
LD_{50} method$_{SC}$									
IV	**−0.53**	**−0.66**	**−0.37**	**−0.52**	**−0.67**	**−0.39**	**−0.52**	**−0.67**	**−0.39**
IP	**−0.26**	**−0.41**	**−0.11**	**−0.25**	**−0.40**	**−0.11**	**−0.25**	**−0.39**	**−0.10**
IM	−0.12	−0.31	0.04	−0.10	−0.27	0.07	−0.10	−0.28	0.07
Eggs in Diet$_{abscent}$									
Present	**0.90**	**0.40**	**1.43**	**0.89**	**0.33**	**1.42**	**0.91**	**0.38**	**1.45**
$D_{LD50\text{-}Diet}$	**0.22**	**0.12**	**0.32**	**0.12**	**0.03**	**0.21**	**0.13**	**0.04**	**0.21**
Diet Breadth$_{Low}$				0.04	−0.05	0.14	0.02	−0.07	0.12
Intermediate	0.08	−0.19	0.34						
High	0.13	−0.14	0.45						
DB_{Low}:$D_{LD50\text{-}Diet}$				−0.06	−0.13	0.02	−0.05	−0.12	0.02
Intermediate	**−0.22**	**−0.41**	**−0.06**						
High	**−0.25**	**−0.50**	**−0.02**						
Constriction$_{absent}$									
Present	−0.14	−0.75	0.42	−0.18	−0.73	0.45	−0.17	−0.81	0.38
**Random Terms**									
Phylogeny (h^2)	0.42	0.18	0.68	0.46	0.21	0.71	0.46	0.21	0.72
Species	0.15	0.01	0.33	0.13	0.01	0.31	0.13	0.01	0.31
Residuals	0.41	0.26	0.54	0.39	0.26	0.52	0.39	0.25	0.52

Table S3. Models with mean prey size included as a fixed factor. Modes (β) and 95% credibility intervals (Lower CI and Upper CI) of the posterior distributions from the three main models predicting LD_{50} using Taxonomic richness, Phylogenetic Diversity and Species Richness as measures of diet breadth. Fixed factors include the continuous factors of mean phylogenetic distance between diet species and the LD_{50} model ($D_{LD50\text{-}Diet}$); mean prey size and Diet Breadth (DB) in models that use Phylogenetic Diversity and Species Richness. Categorical fixed factors include LD_{50} method (subcutaneous (SC), intravenous (IV), intraperitoneal (IP) and intramuscular (IM)); the presence of eggs in the diet (Eggs in Diet; present, absent) and the Taxonomic Richness measure of Diet Breadth. For categorical factors the baseline was SC for LD_{50} method; the absence of eggs for the Eggs in Diet and low for Taxonomic Richness. DB_{Low}:$D_{LD50\text{-}Diet}$ represents the interaction term between DB and $D_{LD50\text{-}Diet}$. The random terms associated with phylogenetic relatedness (Phylogeny (h^2)), intraspecific variation (Species) and residual variation (Residual) are also presented. Significant values, which are highlighted in bold, are deemed to be those with 95% of the posterior estimate above or below zero. All models have 396 LD_{50} measures for 70 species.

	**Taxonomic Richness Model**			**Phylogenetic Diversity Model**			**Species Richness Model**		
	**β**	**Lower CI**	**Upper CI**	**β**	**Lower CI**	**Upper CI**	**β**	**Lower CI**	**Upper CI**
**Fixed Terms**									
Intercept	0.33	−0.23	0.90	0.41	−0.20	0.99	0.40	−0.17	0.96
LD_{50} method$_{SC}$									
IV	**−0.53**	**−0.70**	**−0.36**	**−0.53**	**−0.71**	**−0.36**	**−0.54**	**−0.72**	**−0.37**
IP	**−0.32**	**−0.48**	**−0.13**	**−0.31**	**−0.46**	**−0.13**	**−0.31**	**−0.49**	**−0.14**
IM	−0.14	−0.35	0.06	−0.12	−0.31	0.09	−0.13	−0.33	0.09
Eggs in Diet$_{abscent}$									
Present	**0.75**	**0.18**	**1.32**	**0.71**	**0.11**	**1.26**	**0.73**	**0.14**	**1.31**
$D_{LD50\text{-}Diet}$	**0.27**	**0.16**	**0.38**	**0.14**	**0.04**	**0.25**	**0.17**	**0.07**	**0.27**
Diet Breadth$_{Low}$				0.05	−0.06	0.17	0.04	−0.07	0.15
Intermediate	0.14	−0.13	0.47						
High	0.08	−0.26	0.40						
DB_{Low}:$D_{LD50\text{-}Diet}$				−0.07	−0.17	0.02	−0.06	−0.16	0.02

Intermediate	**−0.30**	**−0.51**	**−0.09**						
High	−0.24	−0.53	0.08						
Mean prey size	0.08	−0.04	0.18	0.06	−0.07	0.18	0.07	−0.06	0.19
**Random Terms**									
Phylogeny (h^2)	0.63	0.33	0.79	0.66	0.29	0.79	0.68	0.32	0.80
Species	0.01	0.01	0.23	0.01	0.01	0.26	0.01	0.01	0.26
Residuals	0.36	0.23	0.51	0.34	0.21	0.50	0.33	0.20	0.48

Table S4. Models with LD_{50} duration included as a fixed factor. Modes (β) and 95% credibility intervals (Lower CI and Upper CI) of the posterior distributions from the three main models predicting LD_{50} using Taxonomic richness, Phylogenetic Diversity and Species Richness as measures of diet breadth. Fixed factors include the continuous factors of mean phylogenetic distance between diet species and the LD_{50} model ($D_{LD50\text{-}Diet}$); the duration of the LD_{50} experiment (LD_{50} Duration) and Diet Breadth (DB) in models that use Phylogenetic Diversity and Species Richness. Categorical fixed factors include LD_{50} method (subcutaneous (SC), intravenous (IV), intraperitoneal (IP) and intramuscular (IM)); the presence of eggs in the diet (Eggs in Diet; present, absent) and the Taxonomic Richness measure of Diet Breadth. For categorical factors the baseline was SC for LD_{50} method; the absence of eggs for the Eggs in Diet and low for Taxonomic Richness. DB_{Low}:$D_{LD50\text{-}Diet}$ represents the interaction term between DB and $D_{LD50\text{-}Diet}$. The random terms associated with phylogenetic relatedness (Phylogeny (h^2)), intraspecific variation (Species) and residual variation (Residual) are also presented. Significant values, which are highlighted in bold, are deemed to be those with 95% of the posterior estimate above or below zero. All models have 315 LD_{50} measures for 82 species.

	**Taxonomic Richness Model**			**Phylogenetic Diversity Model**			**Species Richness Model**		
	**β**	**Lower CI**	**Upper CI**	**β**	**Lower CI**	**Upper CI**	**β**	**Lower CI**	**Upper CI**
**Fixed Terms**									
Intercept	0.06	−0.45	0.63	0.27	−0.21	0.82	0.24	−0.27	0.74
LD_{50} $method_{SC}$									
IV	**−0.54**	**−0.80**	**−0.28**	**−0.53**	**−0.80**	**−0.27**	**−0.52**	**−0.80**	**−0.27**
IP	−0.21	−0.46	0.05	−0.19	−0.46	0.05	−0.17	−0.42	0.12
IM	−0.05	−0.32	0.24	−0.03	−0.31	0.26	−0.01	−0.31	0.27
Eggs in $Diet_{abscent}$									
Present	**0.96**	**0.25**	**1.62**	**0.91**	**0.09**	**1.61**	**0.96**	**0.23**	**1.66**
$D_{LD50\text{–}Diet}$	**0.24**	**0.12**	**0.35**	**0.10**	**0.01**	**0.20**	**0.12**	**0.02**	**0.21**
Diet $Breadth_{Low}$				0.05	−0.07	0.17	0.02	−0.09	0.13
Intermediate	0.27	−0.05	0.59	–	–	–	–	–	–
High	0.32	−0.07	0.69	–	–	–	–	–	–
DB_{Low}:$D_{LD50\text{-}Diet}$				−0.08	−0.17	0.02	−0.07	−0.17	0.02
Intermediate	**−0.28**	**−0.47**	**−0.09**	–	–	–	–	–	–
High	−0.26	−0.55	0.01	–	–	–	–	–	–
LD_{50} Duration	−0.02	−0.09	0.04	−0.02	−0.08	0.04	−0.02	−0.09	0.04
**Random Terms**									
Phylogeny (h^2)	0.32	0.08	0.63	0.35	0.13	0.67	0.38	0.14	0.68
Species	0.23	0.03	0.45	0.21	0.02	0.43	0.21	0.01	0.41
Residuals	0.40	0.26	0.55	0.38	0.24	0.53	0.38	0.23	0.52

Table S5. Models with the wild/captive status of the LD_{50} model included as a fixed factor. Modes (β) and 95% credibility intervals (Lower CI and Upper CI) of the posterior distributions from the three main models predicting LD_{50} using Taxonomic richness, Phylogenetic Diversity and Species Richness as measures of diet breadth. Fixed factors include the continuous factors of mean phylogenetic distance between diet species and the LD_{50} model ($D_{LD50\text{-}Diet}$) and Diet Breadth (DB) in models that use Phylogenetic Diversity and Species Richness. Categorical fixed factors include LD_{50} method (subcutaneous (SC), intravenous (IV), intraperitoneal (IP) and intramuscular (IM)); the presence of eggs in the diet (Eggs in Diet; present, absent); the wild/captive origin of the LD_{50} model (LD_{50} captive, wild captive) and the Taxonomic Richness measure of Diet Breadth. For categorical factors the baseline was SC for LD_{50} method; the absence of eggs for the Eggs in Diet; Captive for LD_{50} captive and low for Taxonomic Richness. DB_{Low}:$D_{LD50\text{-}Diet}$ represents the interaction term between DB and $D_{LD50\text{-}Diet}$. The random terms associated

with phylogenetic relatedness (Phylogeny (h^2)), intraspecific variation (Species) and residual variation (Residual) are also presented. Significant values, which are highlighted in bold, are deemed to be those with 95% of the posterior estimate above or below zero. All models have 492 LD_{50} measures for 100 species.

	Taxonomic Richness Model			Phylogenetic Diversity Model			Species Richness Model		
	**β**	**Lower CI**	**Upper CI**	**β**	**Lower CI**	**Upper CI**	**β**	**Lower CI**	**Upper CI**
**Fixed Terms**									
Intercept	0.33	−0.14	0.77	0.38	−0.07	0.87	0.37	−0.12	0.83
LD_{50} method$_{SC}$									
IV	**−0.43**	**−0.56**	**−0.30**	**−0.43**	**−0.56**	**−0.30**	**−0.43**	**−0.56**	**−0.30**
IP	**−0.30**	**−0.43**	**−0.18**	**−0.30**	**−0.43**	**−0.17**	**−0.30**	**−0.44**	**−0.17**
IM	−0.13	−0.29	0.02	−0.14	−0.29	0.04	−0.14	−0.30	0.04
Eggs in Diet$_{abscent}$									
Present	**0.87**	**0.40**	**1.34**	**0.86**	**0.37**	**1.35**	**0.89**	**0.43**	**1.43**
$D_{LD50\text{-}Diet}$	**0.27**	**0.17**	**0.36**	**0.17**	**0.10**	**0.26**	**0.19**	**0.11**	**0.27**
Diet Breadth$_{Low}$				0.01	−0.07	0.10	0.01	−0.07	0.10
Intermediate	0.05	−0.21	0.29	–	–	–	–	–	–
High	0.11	−0.18	0.40	–	–	–	–	–	–
DB_{Low}:$D_{LD50\text{-}Diet}$				−0.04	−0.10	0.03	−0.02	−0.09	0.05
Intermediate	−0.13	−0.30	0.02	–	–	–	–	–	–
High	**−0.38**	**−0.61**	**−0.14**	–	–	–	–	–	–
LD_{50} captive									
Wild	**0.25**	**0.07**	**0.41**	**0.25**	**0.09**	**0.43**	**0.27**	**0.09**	**0.44**
**Random Terms**									
Phylogeny (h^2)	0.48	0.24	0.73	0.53	0.28	0.77	0.56	0.28	0.78
Species	0.16	0.01	0.34	0.14	0.01	0.30	0.11	0.01	0.30
Residuals	0.35	0.22	0.47	0.33	0.21	0.46	0.31	0.21	0.45

Table S6. Sensitivity analysis of Taxonomic Richness Models. Modes (β) and 95% credibility intervals (Lower CI and Upper CI) of the posterior distributions from the sensitivity analysis models predicting LD_{50} using Taxonomic richness with differing category definitions. Fixed factors include the continuous factors mean phylogenetic distance between diet species and the LD_{50} model ($D_{LD50\text{-}Diet}$). Categorical fixed factors include LD_{50} method (subcutaneous (SC), intravenous (IV), intraperitoneal (IP) and intramuscular (IM)); the presence of eggs in the diet (Eggs in Diet; present, absent) and Taxonomic richness (Diet Breadth, Low, Intermediate and high). The baseline was SC for LD_{50} method, the absence of eggs for the Eggs in Diet and Low for Diet Breadth. For the sensitivity analysis 1 taxonomic richness was split as low (1 taxonomic group present in the diet), intermediate (2–3 taxonomic groups) and high (more than 3 taxonomic groups) and for sensitivity analysis 2 taxonomic richness was split as low (1 taxonomic group), intermediate (2–4 taxonomic groups) and high (more than 4 taxonomic groups). DB_{Low}:$D_{LD50\text{-}Diet}$ represents the interaction term between DB and $D_{LD50\text{-}Diet}$. The random terms associated with phylogenetic relatedness (Phylogeny (h^2)), intraspecific variation (Species) and residual variation (Residual) are also presented. Significant values, which are highlighted in bold, are deemed to be those with 95% of the posterior estimate above or below zero. All models have 529 LD_{50} measures for 100 species.

	Sensitivity Analysis 1			Sensitivity analysis 2		
	β	Lower CI	Upper CI	β	Lower CI	Upper CI
**Fixed Terms**						
Intercept	0.33	−0.11	0.85	0.34	−0.14	0.83
LD_{50} method$_{SC}$						
IV	**−0.53**	**−0.65**	**−0.37**	**−0.53**	**−0.68**	**−0.40**
IP	**−0.27**	**−0.41**	**−0.13**	**−0.26**	**−0.41**	**−0.12**
IM	−0.12	−0.28	0.07	−0.12	−0.29	0.05
Eggs in Diet$_{abscent}$						
Present	**0.89**	**0.36**	**1.38**	**0.89**	**0.38**	**1.39**
$D_{LD50\text{-}Diet}$	**0.22**	**0.12**	**0.32**	**0.22**	**0.11**	**0.33**
Diet Breadth$_{Low}$						

Intermediate	0.07	−0.20	0.30	0.07	−0.17	0.31
High	0.17	−0.29	0.59	0.16	−0.25	0.64
DB_{Low}:$D_{LD50\text{-}Diet}$						
Intermediate	**−0.22**	**−0.37**	**−0.06**	**−0.22**	**−0.39**	**−0.06**
High	**−0.26**	**−0.50**	**−0.01**	−0.25	−0.50	0.01
**Random Terms**						
Phylogeny (h^2)	0.44	0.20	0.68	0.44	0.20	0.68
Species	0.15	0.01	0.32	0.15	0.01	0.31
Residuals	0.40	0.26	0.54	0.39	0.27	0.53
